# Influence of Classroom Acoustics on Noise Disturbance and Well-Being for First Graders

**DOI:** 10.3389/fpsyg.2019.02736

**Published:** 2019-12-13

**Authors:** Arianna Astolfi, Giuseppina Emma Puglisi, Silvia Murgia, Greta Minelli, Franco Pellerey, Andrea Prato, Tiziana Sacco

**Affiliations:** ^1^Department of Energy, Polytechnic University of Turin, Turin, Italy; ^2^Department of Mathematical Sciences, Polytechnic University of Turin, Turin, Italy; ^3^INRiM – National Institute of Metrological Research, Turin, Italy; ^4^Department of Neurosciences, University of Turin, Turin, Italy

**Keywords:** well-being, noise disturbance, classroom acoustics, reverberation, first graders, happiness

## Abstract

Several studies have shown so far that poor acoustics inside classrooms negatively affects the teaching and learning processes, especially at the lowest grades of education. However, the extent to which noise exposure or excessive reverberation affect well-being of children at school in their early childhood is still unanswered, as well as their awareness of noise disturbance. This work is a pilot study to investigate to which extent classroom acoustics affects the perceived well-being and noise disturbance in first graders. About 330 pupils aged from 6 to 7 years participated in the study. They belonged to 20 classes of 10 primary schools located in Torino (Italy), where room acoustic measurements were performed and where noise level was monitored during classes. The school buildings and the classrooms were balanced between socioeconomic status and acoustic conditions. Trained experimenters administered questionnaires in each class, where pupils answered all together during the last month of the school year (May). Questions included the happiness scale, subscales assessing self-esteem, emotional health, relationship at home and with friends, enjoyment of school, intensity and noise disturbance due to different sound sources, and quality of voice. The findings of the study suggest that long reverberation times, which are associated with poor classroom acoustics as they generate higher noise levels and degraded speech intelligibility, bring pupils to a reduced perception of having fun and being happy with themselves. Furthermore, bad classroom acoustics is also related to an increased perception of noise intensity and disturbance, particularly in the case of traffic noise and noise from adjacent school environments. Finally, happy pupils reported a higher perception of noise disturbance under bad classroom acoustic conditions, whereas unhappy pupils only reported complaints in bad classroom acoustics with respect to the perception of pleasances with himself or herself and of fitting in at school. Being a mother tongue speaker is a characteristic of children that brings more chances of attending classes in good acoustics, of being less disturbed, and of having more well-being, and richer districts presented better acoustic conditions, in turn resulting in richer districts also revealing a greater perception of well-being.

## Introduction

According to the (World Health Organisation [WHO], 2014), the physical environment in schools is one of the major elements of health promotion, and among the stressful environmental factors, high levels of noise can cause irritation, encourage aggressiveness, reduce physical and mental performance, and cause discomfort and headaches. Furthermore, children with learning difficulties, which are usually included in regular classes, are particularly dependent on a good acoustic (GA) environment (Winblad and Dudley, 1997).

Research has widely focused so far on the effects that classroom acoustics has on the teaching and learning processes, even at the lowest grades of education, but few studies have investigated the perception of noise disturbance at school and the influence of bad acoustics (BA), i.e., both excessive noise and reverberation, on the pupil’s well-being. Particularly, no study has investigated, with in-classroom surveys, children well-being at school. Another important lack in the literature is the investigation of fundamental aspects of school life at the lowest grade of primary education, i.e., for most of the countries in Europe from 6 to 7 years. It is in the early childhood that the neuroplasticity of the human brain cortex is still high, and interventions can produce more positive effects. At the cortical level, various sensory and cognitive systems interact and adjust functional properties based upon experience and learning (Cardon et al., 2012).

In the achievement-oriented context of a classroom, well-being is a necessary precondition for learning (Hascher, 2008). School well-being is a multidimensional phenomenon in which occur school conditions, social relationships, means for self-fulfillment, and health status (Konu et al., 2002). Moreover, well-being of primary school pupils is positively influenced by learning skills (Epstein and McPartland, 1976; Tobia et al., 2019), which in turn are negatively influenced by BA (Puglisi et al., 2017).

### Effect of Bad Classroom Acoustics on Learning Attainments of Children

On the one side, with very high reverberation time, primary school teachers raise their voice in order to be understood by pupils (Bottalico and Astolfi, 2012; Puglisi et al., 2017). This is mainly due to the effect of amplification of indoor noise due to excessive sound reflection. High noise levels can bring dysphonia or other vocal pathologies for teachers (Astolfi et al., 2012a; Bottalico et al., 2017a, b), which in turn can determine increased listening difficulties for children (Rudner et al., 2018). On the other side, thanks to voice monitoring performed with professional dosimeters (Carullo et al., 2013; Bottalico et al., 2018) and the uncertainty estimation of the vocal parameters (Castellana et al., 2017; Astolfi et al., 2018a), research has shown detrimental effects in speech production either in low reverberation (Astolfi et al., 2019) or in high reverberation (Astolfi et al., 2015) in the absence of noise, and optimal reverberation times for speaking have been proposed (Pelegrín-García et al., 2014; Calosso et al., 2017; Puglisi et al., 2017).

Unfavorable acoustics in classroom determines challenging environments for children, who are more sensitive than adults or older peers to noise and reverberation when performing tasks that involve listening comprehension and non-auditory features such as short-term memory, reading, and writing (Klatte et al., 2013). As a result, BA brings lower speech intelligibility scores, mostly for first graders (Astolfi et al., 2012b; Prodi et al., 2013; Puglisi et al., 2015b); degradation of the accuracy in identifying and producing newly learned words (Riley and McGregor, 2012); reduced reading speed of second graders (Puglisi et al., 2018); and lower scores in the standardized tests of literacy, mathematics, and science for pupils aged 7–11 years (Shield and Dockrell, 2008).

### Children Perception of the Sound Environment at School

Together with the presence of noise sources as distractors for children’s ability to understand, the subjective perception of the sound environment makes it very different the way listeners experience their everyday living spaces. Brännström et al. (2017) investigated on the 9- to 13-year-old children’s personal ratings of perceived noise in order to improve the classrooms’ design. They found that children were more annoyed to noise in tasks where the demands of verbal processing are higher. Dockrell and Shield (2004) administered a survey on the perception of noise in schools to children of grades 2 and 6. The more the external noise level increased, the more they were annoyed and the less they reported to be able to hear the teacher speaking inside the classroom. Astolfi and Pellerey (2008) assessed the subjective and objective environmental quality in classrooms involving 1,006 high school students with an average age of 16.1 years. They found that students reported to be strongly annoyed by noise sources internal in the classroom, i.e., other students talking in the classroom, and as a side effect of poor classroom acoustics on the overall perception of the school environment, students reported a decrease in their ability to concentrate.

#### Association Between Noise Annoyance and Well-Being

Given the above evidences, it is clear that the learning process is affected by the sound environment. Extensive literature on the subjective perception of the sound environment, especially with surveys in-field, is anyway lacking so far on the possible comorbidities that go beyond the students’ performance at school. Particularly, research should thus focus on the assessment of the association between the perception of sound environment at school, in terms of noise annoyance, and of the wide concept of personal well-being. In fact, as the concept of well-being is composed of three main aspects, that is, subjective, psychological, and social well-being (Ryff and Singer, 1998; Diener, 2006; Keyes, 2013), it can be assumed to be strongly linked to the experience of everyday life situations and circumstances.

Some researchers found no association between noise from airplane and traffic and children well-being. In particular, chronic aircraft noise exposure resulted associated with high levels of noise annoyance but not to mental health problems in over 300 children aged 8–11 years (Haines et al., 2001). Mental health is defined by World Health Organisation [WHO] (2014) as “a state of well-being in which every individual realizes his or her own potential, can cope with the normal stresses of life, can work productively and fruitfully, and is able to make a contribution to her or his community.” Also, Stansfeld et al. (2009) reported no association between either aircraft or road traffic noise exposure and the Strengths and Difficulties Questionnaire (SDQ) total score, in more than 2,000 children aged 9–11 years. This questionnaire is a largely used psychometrically valid instrument to assess mental health of children aged 3–16 years, but the drawback of this tool is that for younger children, it has to be filled in by parents at home (Goodman, 2001).

On the opposite, Crombie et al. (2011) showed association between noise and mental health. Particularly, they found a relationship between road- and aircraft-generated noise and the incidence of mental problems in 9- to 10-year-old children. Lim et al. (2018) have found that high noise levels and high noise sensitivity determine less mental health of children and adolescents and that the effects of these noise-related variables depend on socioenvironmental factors. At school, Klatte and Hellbrück (2010) found that the children from classrooms with poor acoustics reported a higher burden of indoor noise in the classrooms and judged their relationships to their peers and teachers less positively than children from classrooms with GA. As a further study, Stansfeld et al. (2000) reviewed a set of studies on the relationship between noise and mental health. They found, as a general result, that noise was associated with stress-related factors of mental health (e.g., self-reported stress, sociability, and behaviors) and with well-being in children.

Other researchers investigated physiological symptoms of mental health, such as Evans et al. (2001) who found that children who lived in noisier areas had elevated resting systolic blood pressure and 8-h overnight urinary cortisol. Moreover, under laboratory conditions, they found that children from noisier neighborhoods showed elevated heart rate reactivity to reading tests. Similarly, Wålinder et al. (2007) studied the physiological and psychological stress reactions of children in relation to classroom noise. They reported that equivalent noise levels in classrooms, in the range between 59 and 87 dB(A), were significantly related to an increased prevalence of symptoms of fatigue and headache and to a reduced diurnal cortisol variability, indicating that noise should be focused on as a risk factor for children’s well-being in the school environment.

As comorbidities, the exposure to road traffic noise in both residential and school areas was found to be associated in children aged between 7 and 11 years with emotional symptoms (Dreger et al., 2015) and hyperactivity and inattention (Forns et al., 2016; Hjortebjerg et al., 2016).

### Need to Investigate the Effect of BA at School on Noise Disturbance and Well-Being for First Graders

Going beyond the available knowledge is therefore necessary, especially to understand if BA and other factors at school, where children spend most of their time, directly influence their harmonic growth, learning, and well-being. The research should be focused at the lowest grades of education, when interventions can be more effective for children. In addition, subjective outcomes should be acquired by children themselves at school, while they are having classes, in order to catch their feeling while they are immersed in the classroom environment. Most of the previous studies on well-being for children aged less than 11 years are instead based on questionnaires administrated to parents or filled in by parents or children at home, and to the authors’ knowledge, only few works are available so far in literature that carried out well-being and noise disturbance surveys with first graders at school. And few studies compared results from questionnaires with acoustic measures of noise, room acoustics, and speech intelligibility. This comparison is essential for planning future interventions and increase learning and well-being of children.

This work is thus a pilot study to investigate to which extent classroom acoustics affects the perceived well-being and noise disturbance in children. Noise disturbance has been used in this study, instead of noise annoyance, as noise annoyance is a multifaceted concept that could be more complicated for children as it includes, beside noise disturbance, interferences with some activities, nuisance, unpleasantness, and other factors (Guski et al., 1999; Di Blasio et al., 2019).

The purposes of the adopted approach, which was based on the combination of objective and subjective measurements, can be summarized in three main points: (i) assessment of classroom acoustics of first graders at school; (ii) assessment of the perceived well-being and noise disturbance at school; and (iii) association between classroom acoustics, perceived well-being, and noise disturbance. To reach the proposed objectives, room acoustic measurements were performed, and noise levels were monitored during classes. Then, based on the work by Sabri et al. (2015), questions on the perceived well-being inside the classroom environment were designed, containing information on emotional well-being (self-esteem, emotional health, and resilience), friends and family (quality of the relationship), satisfaction of school, and life satisfaction. To build a questionnaire oriented to investigating a multidimensional measure of school-related quality of life, based on Dockrell and Shield (2004) and Astolfi and Pellerey (2008), the abovementioned information was integrated with questions on the perceived noise disturbance.

## Materials and Methods

### Participants

During a meeting for the presentation of the research project, teachers and parents have been informed about the project goal and the scientific evidence of the relationship between acoustics and subjective perceptions and well-being. Only pupils with parental consent were involved in this study, resulting in 367 students from 20 first-grade classes belonging to 10 primary schools in Turin. In the classes, a total number of pupils in the range between 8 and 25 was present. Subjects aged 6 represented 62% of the total, while 37% was aged 7 and only 1% was 8 years old. There were more males (54%) than females (45%). The overall sample consisted of 77% Italian mother tongue (MT) pupils, while 23% used other primary languages in their family context (e.g., Romanian, Moroccan, English, German, Spanish, Albanian, and Arabic). Furthermore, some subjects declared to speak a second language besides Italian. Table 1 shows the sociodemographic characteristics of the considered sample. Twenty-seven subjects were excluded from the data analysis because of cognitive or hearing deficits proved by the school administration through an official medical certificate or due to incorrect completion of the questionnaire.

**TABLE 1:** Description of the classrooms considered in terms of students and building features.

**ID**	**Classroom volume (m^3^)**	**Classes**		**Schools**			
		**Male**	**Italian mother**	**Year of**	**Acoustic**	**District real**	**Traffic**
		**pupils (%)**	**tongue (%)**	**construction**	**treatment**	**estate value (€)**	**volume**
A1	194	60	67	1846	Yes	€€€€€	low
A2	261	50	61	1846	Yes	€€€€€	low
A3	283	58	79	1846	Yes	€€€€€	low
B1	203	50	91	1904	Yes	€€€€€	low
C1	123	58	33	1966	No	€	low
D1	255	67	72	1891	No	€€€€	low
D2	252	57	76	1891	No	€€€€	low
E1	236	68	74	1882	No	€€€	medium
E2	236	62	67	1882	No	€€€	medium
F1	279	61	67	1913	No	€€€€	medium
F2	261	56	44	1913	No	€€€€	medium
G1	136	75	75	1975	No	€€€	low
G2	106	58	95	1975	No	€€€	low
H1	133	57	76	1968	No	€€€€	low
H2	132	48	87	1968	No	€€€€	low
H3	140	50	90	1968	No	€€€€	low
H4	132	50	85	1968	No	€€€€	low
I1	237	13	100	1909	No	€€€	low
L1	241	45	86	1921	No	€€€	low
L2	264	52	96	1921	No	€€€	low

*Schools were identified based on their neighborhood quality [i.e., “district real estate value,” from Turin Real Estate Market Observatory (OICT, 2019) 2019, which was € for the property value interval of €1.000–1.500/m^2^; €€ for €1.500–2.000/m^2^; €€€ for €2.000–2.500/m^2^; €€€€ for €2.500–3.000/m^2^; and €€€€€ for €3.000–3.500/m^2^], the presence of acoustic treatment, the year of construction, and the traffic volume. IDs refer to schools (capital letters) and classrooms (number).*

### Schools and Classrooms

Table 1 reports information about the school buildings and classes involved in the research project. Schools differed in location, period of construction, and architectural features. They are scattered in the city of Turin, in neighborhoods characterized by “low” or “medium” volume of traffic depending on the road typology as defined by the UK Department of Transport (2012), i.e., local road for local traffic or road intended to connect different city areas but not intended to provide large-scale transport links. In the case of medium volume of traffic, classrooms were not directly facing the road, but a courtyard or a corridor was present in between. In the case of low volume of traffic, most of the classrooms faced the road. The presence or absence of an acoustic treatment (AT) in the classrooms (“yes” or “no”) was based on the presence or absence of whatever sound-absorbing material.

### Acoustic Measurements

#### Acoustic Parameters and Adopted Protocol

Acoustic measurements were performed within 1 day approximately in the last 2 months of the school year. Measurements were carried out under occupied conditions, with the number of children inside being on average 18 across all classes. The adopted protocol regarded the acquisition of acoustic parameters that are useful to characterize the classroom’s response to easy listening (Minelli et al., 2019). Below, the equipment, the parameters, and their measurement procedures are described.

**FIGURE 1:**
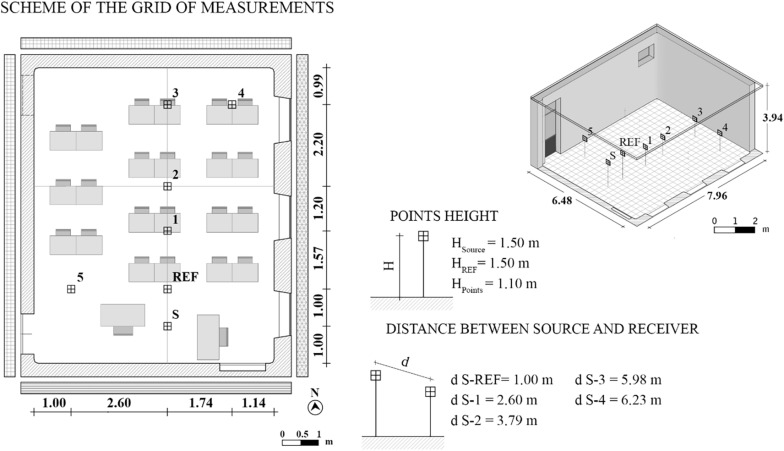
Measurement setup in a typical classroom.

Figure 1 shows the standard measurement setup used in each classroom. All the classrooms showed a traditional distribution of the pupils over the seating area, with the teacher’s desk parallel to one of the shorter sides of the room, so that the source position S has been chosen and several microphone positions were considered. In particular, a fixed reference position that was common across all the classrooms was selected, being placed at 1 m from the source and at the same height of the source, i.e., 1.5 m from the ground, and that was named REF. With this microphone position being the same for every classroom, this measure well describe the difference across classrooms of changes in the vocal output, expressed as A-weighted equivalent sound pressure level measured at 1 m from the speaker’s mouth (ISO 9921, 2015), due to the reverberant sound field. Then, positions 1, 2, 3, 4, and 5 were selected to cover the whole seating area, with a fixed height of 1.1 m from the ground and with varying distances from the source depending on the geometrical characteristics of the classrooms. As a source, an acoustic stimuli generator, namely, a TalkBox (by NTi Audio, Schaan, Liechtenstein), which has the directivity pattern of the human voice, was positioned in the representative place that is typically covered by the teacher depending on the classroom’s dimension and distribution, at the height of 1.5 m from the floor. With the aim of acquiring acoustic stimuli for the extraction of several parameters, a calibrated class 1 sound level meter (SLM, model XL2 by NTi Audio, Schaan, Liechtenstein) was located 1.5 m from the floor in the case of the REF position and 1.1 m from the floor for the other microphone positions, which were distributed over the pupils’ seating area. Overall, both the instruments were positioned at least at a distance of 1 m from any surface.

Reverberation time (T20, s) and speech clarity (C50, dB) were measured according to ISO 3382-2 (2008) and ISO 3382-1 (2009), respectively. Room impulse responses were acquired from three repeated exponential sine sweep signals, which were emitted by the TalkBox and recorded by the SLM at each measurement point. The sweep signals were generated with a sample frequency of 44.1 kHz and a resolution of 16 bits. They were designed to cover a range of 0.05–2 kHz and to have a duration of 3 s each, based on the assumption that in cases with moderate background noise, as in the classrooms under study, it is normally a safe practice to use sweeps with a length of two to four times the expected longest reverberation time (ISO 18233, 2006). Particularly, the SLM was moved along the main axes represented in Figure 1 in the receiver positions REF, 1, 2, 3, and 6. Frequency averages were calculated according to Din 18041 (2016) in the range 0.25–2 kHz for T20 and according to ISO 3382-1 (2009) in the range 0.5–1 kHz for C50. A range of optimal occupied T20 was set between 0.5 and 0.8 s, according to a number of recent studies (0.7 s in Yang and Bradley, 2009; 0.8 s in Bottalico and Astolfi, 2012; 0.5–0.6 s in Pelegrín-García et al., 2014; 0.7 s in Puglisi et al., 2017; and 0.8 s in Calosso et al., 2017), which concerned both speech and listening performance in typical primary and secondary school classrooms. Therefore, for the subsequent analyses, classes were split into GA and BA whether they were in or out of such optimal range, respectively. As far as C50 is concerned, it represents an index related to speech intelligibility in the classroom in the presence of low noise. As given in Bradley (1986), it is assumed that an optimal value of C50 for small classrooms with an optimal reverberation time of 0.8 s at middle frequencies should be greater than around 3 dB.

T20 in unoccupied conditions, T20_e, where the subscript “e” is for empty, was measured with a wooden clapper, i.e., two wooden boards hinged together, according to the ISO 3382-2 (2008), as described in Puglisi et al. (2017). The measurement method in the empty condition was different compared to the one in the occupied condition; however, the two procedures can be considered equivalent in the frequency range of interest as described in Puglisi et al. (2018). Optimal values of T20_e, which are proportional to the classroom volume, were derived from Italian technical standard UNI 11367 (2010) as an average value between 0.5 and 1 kHz.

Background noise level (L_N_, dBA) was considered in terms of indoor A-weighted equivalent sound pressure level (L_Aeq_). Repeated measurements were performed based on 3-min acquisitions (Puglisi et al., 2015a), with windows and doors closed to ensure that no external unforeseen noises influenced them, except the typical ones related to traffic and roads. For this measure, the SLM was located in two or three positions in each classroom, which alternatively corresponded to positions 2, 4, and 5, as presented in Figure 1. Noise measurements were carried out with children in silence, L_N_sil_, and with the children performing group activities, L_N_gr_, in order to be representative of typical classroom scenarios. Both the mentioned conditions were guaranteed with the help of the teacher who asked the children to keep silent and then to speak as they were in a traditional group lesson. According to Shield and Dockrell (2008) and BB93 (WSP, 2015), the L_N_sil_ recommended value must be less than or equal to 35 dBA.

The speech signal (L_S_, dBA) was measured to characterize the propagation of a voice signal in each classroom. The TalkBox was positioned in S according to Figure 1, and then it was set to emit a voice signal with a “normal” vocal effort, i.e., corresponding to 60 dBA at 1 m in anechoic conditions to comply with ANSI S3.5 (1997). The speech signals were acquired as an equivalent A-weighted continuous sound pressure level at the SLM, having the source switched on for the receiving positions REF, 1, 2, 3, and 4.

The ratio of useful to detrimental energy (U50, dB) was calculated to consider both the effect of the acoustics of the environment and the effect of the signal-to-noise ratio on speech intelligibility (Bradley et al., 1999). It is obtained in terms of ratio between useful and detrimental energy, i.e., noise and reverberation. Particularly, it is obtained for each position, from C50, L_S_, and L_N_sil_ values. U50 was then averaged between 500 and 1,000 Hz (U_50__.__0__.__5__–__1 kHz_), as it is derived from a C50 value that exhibits the same span of frequency averaging. As given in Bradley et al. (1999), it is assumed that an optimal value of U50 for small classrooms should be greater than 1 dB.

Overall, the acoustic parameters that are distance dependent (i.e., C50, L_S_, and U50) were measured point by point as described above, but then the acquired measures were processed to have single values that are useful for an effective comparison across classes. In particular, for C50 and U50, the values measured across the classroom were averaged together to have a spatial mean, excluding the reference point (REF in Figure 1), being hereafter reported to as “M” in the parameters’ symbols. Such a value was found to be not so different from the central value, i.e., the value measured at position 2 in Figure 1, which is hereafter reported as “ctr” as subscript for C50 and U50, as underlined in Puglisi et al. (2018). Furthermore, L_S_ values, which were measured on the axis in front of S (i.e., acquisitions in points REF, 1, 2, and 3) were associated to obtain its slope per double distance (in decibels per double distance) (Astolfi et al., 2008), which is hereafter referred to as “m” in the parameters symbols.

#### Measurement Results

Table 2 shows the acoustic parameters grouped for GA and BA, i.e., with occupied reverberation time in or out of the optimal range of 0.5–0.8 s, respectively. A good agreement is shown between occupied, T20, and unoccupied reverberation times, T20_e, even though in the case of the unoccupied condition, optimal values are too low compared to the measured values. For the classrooms A2, A3, C1, E1, and I1, T20_e is surprisingly lower or equal to T20. This strange behavior is due to the different frequency range assumed for the averaging, which is only confined to 0.5–1 kHz in the case of the empty room.

**TABLE 2:** Acoustic parameters for classrooms grouped for BA and GA. L_N_sil_ is the noise acquired with students in silence, L_N_gr_ is the noise acquired with students doing group activities; L_S_REF_ is the signal measured at the reference point.

**ID**	**Acoustic parameters**									
	**T20_e (s)**	**T20 (s)**	**L_N_sil_ (dBA)**	**L_N_gr_ (dBA)**	**L_S_REF_ (dBA)**	**mL_S_ (dBA/dd)**	**C50_ctr (dB)**	**C50_M (dB)**	**U50_ctr (dB)**	**U50_M (dB)**
**Bad acoustics**										
A1	1.0 (0.04)	0.9 (0.04)	51.7	NA	61.3	−1.9	1.0	1.3 (1.2)	−1.1	−1.2 (1.0)
A2	**0.8** (0.06)	0.9 (0.18)	49.0	64.7	61.2	−2.4	0.0	2.2 (1.8)	−1.3	0.2 (1.1)
D1	1.4 (0.06)	1.2 (0.31)	51.2	68.0	63.0	−1.8	−0.6	0.0 (0.9)	−2.1	−1.5 (1.0)
D2	1.4 (0.01)	1.3 (0.25)	52.0	NA	62.7	−2.1	0.0	−0.3 (1.1)	−1.6	−1.8 (1.4)
E1	1.2 (0.06)	1.2 (0.11)	54.0	66.6	62.1	−1.4	0.7	1.1 (0.9)	−1.5	−1.2 (0.6)
E2	1.0 (0.01)	0.9 (0.08)	54.3	73.7	61.5	−1.9	3.8	2.7 (1.0)	0.0	−0.9 (0.8)
F1	1.5 (0.01)	1.2 (0.12)	52.0	75.1	62.1	−1.7	1.1	−0.3 (1.8)	−0.9	−2.2 (1.6)
F2	1.7 (0.03)	1.4 (0.33)	52.0	73.8	62.9	−1.8	−1.1	−0.1 (1.2)	−2.7	−1.8 (1.3)
G1	1.2 (0.07)	0.9 (0.05)	51.5	72.2	62.3	−2.1	3.3	2.6 (1.0)	1.3	0.9 (0.9)
I1	1.3 (0.07)	1.4 (0.07)	45.7	59.9	61.9	−1.6	−2.2	−2.2 (0.2)	−2.6	−2.6 (0.2)
L1	NA	1.0 (0.05)	47.9	67.4	61.1	−1.9	1.2	1.6 (1.0)	0.0	0.4 (1.1)
L2	1.2 (0.02)	1.1 (0.07)	46.0	71.6	62.0	−2.2	0.6	0.5 (2.0)	−0.1	−0.2 (2.1)
**Good acoustics**										
A3	**0.8** (0.05)	**0.8** (0.05)	38.4	61.8	60.3	−2.0	**5.1**	**4.1** (0.9)	**4.8**	**3.8** (0.9)
B1	**0.6** (0.07)	**0.5** (0.05)	49.3	66.3	60.8	−2.1	**7.3**	**7.6** (1.5)	**2.9**	**4.0** (1.9)
C1	0.8 (0.03)	**0.8** (0.12)	49.3	62.2	62.8	−1.6	2.8	**3.3** (0.8)	**1.9**	**2.2** (0.6)
H1	0.8 (0.03)	**0.7** (0.20)	51.6	71.9	61.5	−1.1	**3.8**	**3.6** (0.2)	**1.4**	**1.5** (0.2)
H2	0.9 (0.04)	**0.6** (0.08)	55.9	68.1	62.4	−2.2	**5.4**	**5.3** (0.2)	−1.0	−0.8 (0.5)
H3	1.0 (0.03)	**0.7** (0.04)	45.5	63.9	62.9	−1.8	**3.8**	**3.8** (0.3)	**3.0**	**3.2** (0.3)
H4	0.8 (0.04)	**0.7** (0.07)	53.1	65.5	62.9	−2.1	**3.5**	**4.1** (0.6)	−0.1	0.6 (0.6)
G2	0.9 (0.01)	**0.6** (0.01)	51.9	65.3	60.7	−0.8	**3.5**	2.9 (0.9)	**1.4**	0.8 (0.7)

*The slope of a parameter per double distance is referred to with “m” (e.g., mL_*S*_); the mean distribution of a parameter in the classroom is referred to with “M” (e.g., U50_M); the value of a parameter measured in the center of the classroom is referred to with “ctr” (e.g., U50_ctr). Standard deviations are indicated in brackets when available. Not available measures are marked as NA. Values in bold are compliant with the optimal values in occupied conditions.*

Optimal values are shown for speech intelligibility expressed by the parameters C50 and U50, in the classroom with GA, as well as lower values of noise level during group activity, L_N_gr_, that is strictly related to reverberation time. In contrast, noise level in silence, L_N_sil_, does not show decreased values in GA.

No such clear tendency is shown for the parameters related to the speech level, L_S___REF_ and mL_S_, with reverberation time, even though the expected values should be lower in GA and higher in BA. In the case of the slope of the speech signal, mL_S_, higher values (or lower if the slope is taken in absolute value) mean values closer to zero, i.e., absence of slope due to a more uniform acoustic field.

### Questionnaires

Two questionnaires have been designed in order to evaluate children subjective perception of well-being and noise disturbance while in classroom. In both cases, questions have been adapted for children of 6 years old, taking into account readability, comprehension and ease of administration of the document. An Italian translation of the questionnaire developed by Sabri et al. (2015), suitable for young people with special educational needs (SENs), was done and then used as the questionnaire for perceived well-being assessment, while the questionnaire on noise disturbance was adapted on the base of the work by Dockrell and Shield (2004) and by Astolfi and Pellerey (2008). The translation of instructions and items was made using a back-to-back translation, that is, this procedure was used to validate that the translation content did not deviate significantly from the original. Initially, the English version was translated into Italian, then it was translated back to English and this back translation checked with the original for inconsistencies. The back translation was judged to be consistent with the original English version.

The final version of the whole questionnaire is shown in Table 3. It was administrated inside the classrooms where the didactic activity takes place every day, while researchers of the Polytechnic of Turin and teachers assisted the compilation: in particular each question was read by a child in turn who was then asked to explain its meaning. If it was unclear or not understood by all, an intervention by the teacher was required in order to clarify the question. Questionnaires were filled in during sessions of about 40 min each, with a break in between, along 1 day either in May 2017 or in May 2018. Approval to conduct the present study was granted by the Politecnico di Torino Ethics Committee.

**TABLE 3:** Administered questionnaires on the perceived well-being and noise disturbance, with scales and labels question by question.

**Topic**	**ID**	**Questions**	**Scale**	**Labels**
**Well-being**				
Self-esteem	Q1_WB	I am proud of myself	1–3	Yes (1) Not sure (2) No (3)
	Q2_WB	I’m serene	1–3	
	Q3_WB	I have a lot of fun	1–3	
Emotional health	Q4_WB	Lots of things about me are good	1–3	
	Q5_WB	I feel pleased with myself	1–3	
	Q6_WB	I am a cheerful child	1–3	
Relationship at home and with friends	Q7_WB	My home is a good place to relax	1–3	
	Q8_WB	I enjoy myself with my friends	1–3	
	Q9_WB	My friends help me if I need it	1–3	
Enjoyment of school	Q10_WB	I like being in school	1–3	
	Q11_WB	I feel safe at school	1–3	
	Q12_WB	I feel like I fit in at school	1–3	
Scale of happiness	Q13_WB	Put a cross on the number corresponding to your degree of happiness	0–10	Not happy (0) Happy (10)
**Noise**				
Traffic disturbance	Q1_N	How much traffic noise disturb you?	1–3	A little (1) Quite (2) A lot (3)
Siren disturbance	Q2_N	How much ambulance, firemen and police sirens disturb you?	1–3	
Disturbance from internal noises	Q3_N	How much sounds of radios or recorders coming from other classrooms or from the corridor disturb you?	1–3	
Rain disturbance	Q4_N	How much rain noise, if it’s raining outside, disturb you?	1–3	
Intensity and disturbance during silent task	Q5_N	The noise present when you perform a task in silence seems you.	1–3	Low (1) Medium (2) High (3)
	Q6_N	How much does this noise disturb you?	1–3	A little (1) Quite (2) A lot (3)
Intensity and disturbance during group activity	Q7_N	The noise present when you’re working in a group seems to you.	1–3	Low (1) Medium (2) High (3)
	Q8_N	How much does this noise disturb you?	1–3	A little (1) Quite (2) A lot (3)
Quality of voice	Q9_N	How do you hear the teacher’ voice if you are in silence while she is talking?	1–3	Good (1) Quite good (2) Bad (3)
	Q10_N	How do you hear your classmate’s voice if he or she is answering to the teacher?	1–3	

The well-being questionnaire started with an introductory section that consists in five items on sociodemographic information such as age, gender, number of people living at home, the quietest place known by the individual, primary language spoken in family. Finally, the last item of the section and the questionnaire is an open question where the child is asked to report an opinion about the feelings of their school sound environment. Then the questionnaire presents five sections: (1) self-esteem; (2) emotional health; (3) relationship at home and with friends; (4) enjoyment of school; (5) scale of happiness. Sections 1–4 consist in three questions each, where a three-point ordinal scale allows to choose the accordance among the options (a) yes, (b) not sure or (c) no. For section 5 the evaluation scale consists in a 11-point scale, where pupils had to put a cross on the number of an illustrated stair corresponding to their perceived level of happiness. A visual feedback with sketches and emoticons helped in the compilation of the questionnaire.

The noise questionnaire contained three sections: (1) perceived disturbance of specific noise sources (i.e., traffic, car sirens, internal noise, and natural noise), (2) perceived intensity and disturbance of noise during school activities performed either in silence or in group, (3) perceived voice quality under two situations, that is, while a classmate asks a question or while the teacher explains. The described sections were associated with three-point ordinal scales of evaluation, in which the judging items typically varied from the less (left) to the most (right) disturbing/annoying response. As in the well-being questionnaire, figures and emoticons were used to make it easier to identify the type of noise being investigated and a symbology that facilitated the indication of the perceived disturbance. Finally, the last item of the questionnaire consisted in an open question where the child was asked to optionally add comments.

### Statistical Analysis

The statistical analysis was carried out with SPSS (IBM Statistics 20, IBM, Armonk, NY, United States). In order to detect and eliminate outliers from the original sample, two different methodologies have been applied, one that refers to the well-being answers and the other based on the noise answers. As far as the well-being answers, pupils have been divided in two groups based on the Q13_WB answer, i.e., the happiness scale, through a 2-means cluster analysis. Particularly, unhappy children have been considered for answers from 0 to 6, and happy children for answers from 7 to 10. Then, a logistic regression has been carried out considering the membership in the group as the response variable and the others well-being answers as explicative variables, and the Cook’s distance, for every pupil has been obtained. The Cook’s distance measures inconsistence between the response variables and the explicative ones. All the pupils with a Cook’s distance higher than 0.15 have been recognized as outliers. As far as the noise answers is concerned, outliers have been automatically recognized for each class, by the corresponding box-plots related to the means of the answers from Q1_N to Q10_N.

In the current sample, the Cronbach’s α values of 0.69 and 0.71 have been obtained from the answers to the two questionnaires on well-being and noise disturbances, respectively, thus showing the internal consistency of both the questionnaires, i.e., a good intercorrelations among test items in each set of questions. But, being the two values not sufficiently close to unity, they also reveal that the two sets of items actually measure several unrelated latent constructs. This fact confirms that both questionnaires are suitable to capture different aspects of well-being and of noise disturbances, in the form they were designed.

Non-parametric methods have been used to analyze data obtained with ordinal scales, as in the case of this study (Sigel and Castellan, 1988). The significance of the differences between happy and unhappy children in good and bad classroom acoustics, related to several factors concerning well-being and noise, as well as the differences between males and females, was assessed with the Mann–Whitney *U* Test (MWU), a test that is used for two groups of independent observations.

The relationships between the subjective outcomes and the issues concerning the school context, the MT percentage and the classrooms characteristic acoustic parameters, as well as between the well-being and noise disturbance scores and the acoustic parameters, were also investigated through the non-parametric and non-linear correlation estimator Spearman’s rho (Croux and Dehon, 2010).

In order to perform a more robust correlation analysis, the acoustic parameters have been previously analyzed with respect to their expected relationships with the parameter Reverberation time, T20. All the acoustic parameters for each classroom, except L_Aeq_sil_, have been related to T20 through a linear regression, and the classrooms that did show evident anomalous tendencies have been considered outliers and thus canceled from the database. From zero to three classroom values for each parameter have been canceled from the original database. The anomalies were due to non-uniform reverberant sound field in the classroom caused by concentration due to vaulted ceilings, shape of the room, causalities in the measurements and so on.

## Results

After the removal of outliers, a final sample of 326 questionnaires was used for the subjective analyses. The students were almost equally subdivided between males (54%) and females (46%), and 79.0% were Italian. A reduced sample of 296 students, corresponding to the happy children, were also used for the correlation between the subjective and objective acoustic data and between the subjective data and the classes and schools’ characteristics. Unhappy children have been removed from this analysis in order to have a more homogeneous sample.

A preliminary analysis has been made in order to get differences between males and females on the single questions, considering the whole sample of children after the removal of the outliers. Apart from the questions Q5_WB (pleasances with himself or herself) and Q6_WB (cheerfulness), for which a statistical significant difference has been found between males and females according to the MWU test, and lower average scores have been gathered by males for both the questions, no difference has been found for the other well-being and noise disturbance aspects. Females have been found less pleased with themselves and less cheerful than males.

The main statistical analyses have then been carried out and the results commented in the paragraphs below. In particular, the research questions were explained through the following outcomes:

• Comparison between subjective responses on well-being and noise disturbance in good and bad classroom acoustics for all the students (Table 4), for happy students (Table 5) and for unhappy students (Table 6);

**TABLE 4:** All students’ subjective responses on the perceived well-being and noise disturbance, with the sample being grouped per classroom acoustics (i.e., good and bad).

**Topic**	**ID**	**Scale**	**Good acoustics**			**Bad acoustics**			**MWU *p*-value**
			**(*N* = 139)**			**(*N* = 187)**			
			***M***	**SD**	**95% CI**	***M***	**SD**	**95% CI**	
**Well-being**									
Self-esteem	Q1_WB	1–3	1.24	0.50	1.15–1.32	1.25	0.55	1.17–1.33	0.86
	Q2_WB	1–3	1.43	0.71	1.32–1.55	1.56	0.76	1.45–1.67	0.11
	Q3_WB	1–3	1.19	0.52	1.11–1.28	1.23	0.54	1.16–1.31	0.35
Emotional health	Q4_WB	1–3	1.30	0.55	1.21–1.39	1.34	0.60	1.26–1.43	0.64
	Q5_WB	1–3	1.21	0.47	1.13–1.29	1.32	0.61	1.23–1.41	0.12
	Q6_WB	1–3	1.20	0.50	1.12–1.28	1.24	0.55	1.16–1.32	0.56
Relationship at home and with friends	Q7_WB	1–3	1.28	0.59	1.18–1.38	1.28	0.62	1.19–1.37	0.72
	Q8_WB	1–3	1.12	0.34	1.06–1.17	1.16	0.46	1.09–1.22	0.70
	Q9_WB	1–3	1.32	0.63	1.22–1.43	1.30	0.56	1.23–1.38	0.86
Enjoyment of school	Q10_WB	1–3	1.25	0.56	1.15–1.34	1.41	0.75	1.30–1.51	0.09
	Q11_WB	1–3	1.41	0.65	1.30–1.52	1.40	0.63	1.31–1.49	0.91
	Q12_WB	1–3	1.26	0.56	1.16–1.35	1.42	0.70	1.32–1.53	**0.03**
Scale of happiness	Q13_WB	0–10	9.24	1.83	8.94–9.55	9.28	1.99	9.00–9.57	0.51
**Noise**									
Traffic disturbance	Q1_N	0–3	1.88	0.87	1.65–2.10	1.89	0.84	1.67–2.11	0.92
Siren disturbance	Q2_N	0–3	1.90	0.83	1.72–2.08	1.88	0.78	1.72–2.05	0.93
Disturbance from internal noises	Q3_N	0–3	1.83	0.81	1.66–2.00	1.75	0.75	1.60–1.90	0.56
Rain disturbance	Q4_N	0–3	1.72	0.74	1.57–1.88	1.83	0.91	1.67–1.99	0.58
Intensity and disturbance during silent task	Q5_N	1–3	1.42	0.67	1.31–1.53	1.53	0.74	1.42–1.64	0.18
	Q6_N	1–3	1.28	0.57	1.19–1.38	1.52	0.76	1.41–1.63	**0.01**
Intensity and disturbance during group activity	Q7_N	1–3	1.93	0.80	1.79–2.06	2.14	0.77	2.03–2.25	**0.02**
	Q8_N	1–3	1.74	0.78	1.61–1.88	1.97	0.83	1.85–2.09	**0.02**
Quality of voice	Q9_N	1–3	1.29	0.61	1.19–1.40	1.25	0.55	1.17–1.33	0.65
	Q10_N	1–3	1.58	0.71	1.46–1.70	1.53	0.74	1.43–1.64	0.37

*Mean (*M*), standard deviation (SD) and the 95% confidence interval (95% CI) values are provided, and the *p*-values of significance for the differences between the two acoustic conditions, according to the MWU Test. Any statistically significant differences, with *p*-value < 0.05, are reported in bold.*

**TABLE 5:** Happy students’ subjective responses on the perceived well-being and noise, with the sample being grouped per classroom acoustics (i.e., good and bad).

**Topic**	**ID**	**Scale**	**Good acoustics**			**Bad acoustics**			**MWU *p*-value**
			**(*N* = 124)**			**(*N* = 172)**			
			***M***	**SD**	**95% CI**	***M***	**SD**	**95% CI**	
**Well-being**									
Self-esteem	Q1_WB	1–3	1.22	0.49	1.13–1.30	1.22	0.53	1.16–1.91	0.80
	Q2_WB	1–3	1.43	0.73	1.30–1.56	1.50	0.73	1.81–2.59	0.31
	Q3_WB	1–3	1.14	0.45	1.06–1.22	1.19	0.48	1.24–2.19	0.15
Emotional health	Q4_WB	1–3	1.26	0.52	1.17–1.35	1.33	0.59	1.21–1.86	0.35
	Q5_WB	1–3	1.22	0.49	1.13–1.30	1.28	0.57	1.29–2.18	0.35
	Q6_WB	1–3	1.10	0.31	1.05–1.16	1.15	0.41	1.82–2.71	0.43
Relationship at home and with friends	Q7_WB	1–3	1.23	0.54	1.14–1.33	1.24	0.57	1.25–2.22	0.89
	Q8_WB	1–3	1.09	0.31	1.03–1.14	1.12	0.39	1.18–2.02	0.68
	Q9_WB	1–3	1.27	0.57	1.17–1.37	1.24	0.47	1.53–2.47	0.77
Enjoyment of school	Q10_WB	1–3	1.22	0.53	1.12–1.31	1.35	0.70	1.55–2.59	0.14
	Q11_WB	1–3	1.31	0.59	1.20–1.41	1.30	0.54	2.21–2.73	0.81
	Q12_WB	1–3	1.23	0.52	1.13–1.32	1.33	0.61	2.20–3.00	0.11
**Noise**									
Traffic disturbance	Q1_N	0–3	1.87	0.87	0.62–1.01	1.94	0.83	0.00–0.44	0.65
Siren disturbance	Q2_N	0–3	1.86	0.84	0.86–1.25	1.90	0.77	0.00–0.94	0.68
Disturbance from internal noises	Q3_N	0–3	1.79	0.83	0.93–1.31	1.74	0.76	0.46–1.54	0.78
Rain disturbance	Q4_N	0–3	1.74	0.75	0.92–1.29	1.84	0.91	1.11–2.17	0.67
Intensity and disturbance during silent task	Q5_N	1–3	1.38	0.63	1.27–1.49	1.49	0.72	1.49–2.38	0.19
	Q6_N	1–3	1.26	0.56	1.16–1.36	1.50	0.76	1.33–2.14	**0.01**
Intensity and disturbance during group activity	Q7_N	1–3	1.91	0.80	1.77–2.05	2.12	0.79	2.14–2.66	**0.03**
	Q8_N	1–3	1.72	0.79	1.58–1.86	1.94	0.83	1.86–2.67	**0.03**
Quality of voice	Q9_N	1–3	1.27	0.59	1.16–1.37	1.23	0.53	1.14–1.79	0.74
	Q10_N	1–3	1.50	0.65	1.39–1.62	1.52	0.73	1.28–2.15	0.78

*Mean (*M*), standard deviation (SD) and the 95% confidence interval (95% CI) values are provided, and the *p*-values of significance for the differences between the two acoustic conditions, according to the MWU Test. Any statistically significant differences, with *p*-value < 0.05, are reported in bold.*

**TABLE 6:** Unhappy students’ subjective responses on the perceived well-being and noise disturbance, with the sample being grouped per classroom acoustics (i.e., good and bad).

**Topic**	**ID**	**Scale**	**Good acoustics**			**Bad acoustics**			**MWU *p*-value**
			**(*N* = 15)**			**(*N* = 15)**			
			***M***	**SD**	**95% CI**	***M***	**SD**	**95% CI**	
**Well-being**									
Self-esteem	Q1_WB	1–3	1.40	0.63	1.08–1.72	1.53	0.74	1.16–1.91	0.64
	Q2_WB	1–3	1.47	0.64	1.14–1.79	2.20	0.77	1.81–2.59	**0.01**
	Q3_WB	1–3	1.67	0.82	1.25–2.08	1.71	0.91	1.24–2.19	0.96
Emotional health	Q4_WB	1–3	1.67	0.62	1.35–1.98	1.53	0.64	1.21–1.86	0.52
	Q5_WB	1–3	1.13	0.35	0.96–1.31	1.73	0.88	1.29–2.18	**0.03**
	Q6_WB	1–3	2.00	0.93	1.53–2.47	2.27	0.88	1.82–2.71	0.54
Relationship at home and with friends	Q7_WB	1–3	1.67	0.82	1.25–2.08	1.73	0.96	1.25–2.22	0.96
	Q8_WB	1–3	1.33	0.49	1.09–1.58	1.60	0.83	1.18–2.02	0.47
	Q9_WB	1–3	1.80	0.86	1.36–2.24	2.00	0.93	1.53–2.47	0.55
Enjoyment of school	Q10_WB	1–3	1.50	0.76	1.10–1.90	2.07	1.00	1.55–2.59	0.12
	Q11_WB	1–3	2.27	0.46	2.04–2.50	2.47	0.52	2.21–2.73	0.26
	Q12_WB	1–3	1.50	0.76	1.10–1.90	2.62	0.77	2.20–3.00	**0.00**
**Noise**									
Traffic disturbance	Q1_N	0–3	2.00	1.00	0.87–3.00	1.00	0.00	/	0.12
Siren disturbance	Q2_N	0–3	2.22	0.67	1.79–2.66	1.50	1.00	0.52–2.48	0.14
Disturbance from internal noises	Q3_N	0–3	2.09	0.54	1.77–2.41	1.88	0.64	1.43–2.32	0.42
Rain disturbance	Q4_N	0–3	1.56	0.73	1.08–2.03	1.77	0.93	1.27–2.27	0.66
Intensity and disturbance during silent task	Q5_N	1–3	1.73	0.88	1.29–2.18	1.93	0.88	1.49–2.38	0.52
	Q6_N	1–3	1.47	0.64	1.14–1.79	1.73	0.80	1.33–2.14	0.36
Intensity and disturbance during group activity	Q7_N	1–3	2.07	0.80	1.66–2.47	2.40	0.51	2.14–2.66	0.24
	Q8_N	1–3	1.93	0.70	1.58–2.29	2.27	0.80	1.86–2.67	0.21
Quality of voice	Q9_N	1–3	1.53	0.74	1.16–1.91	1.47	0.64	1.14–1.79	0.89
	Q10_N	1–3	2.20	0.86	1.76–2.64	1.71	0.83	1.28–2.15	0.13

*Mean (*M*), standard deviation (SD), and the 95% confidence interval (95% CI) values are provided and the *p*-values of significance for the differences between the two acoustic conditions, according to the MWU test. Any statistically significant differences, with a *p*-value < 0.05, are reported in bold.*

• Comparison between subjective responses on well-being and noise disturbance for happy and unhappy students in classrooms with GA (Table 7) and BA (Table 8);

**TABLE 7:** Subjective responses on the perceived well-being and noise disturbance of students in good acoustics only, with the sample being grouped per happiness (i.e., happy and unhappy).

**Topic**	**ID**	**Scale**	**Happy subjects**			**Unhappy subjects**			**MWU *p*-value**
			**(*N* = 124)**			**(*N* = 15)**			
			***M***	**SD**	**95% CI**	***M***	**SD**	**95% CI**	
**Well-being**									
Self-esteem	Q1_WB	1–3	1.22	0.49	1.13–1.30	1.40	0.63	1.08–1.72	0.18
	Q2_WB	1–3	1.43	0.73	1.30–1.56	1.47	0.64	1.14–1.79	0.58
	Q3_WB	1–3	1.14	0.45	1.06–1.22	1.67	0.82	1.25–2.08	**0.00**
Emotional health	Q4_WB	1–3	1.26	0.52	1.17–1.35	1.67	0.62	1.35–1.98	**0.00**
	Q5_WB	1–3	1.22	0.49	1.13–1.30	1.13	0.35	0.96–1.31	0.59
	Q6_WB	1–3	1.10	0.31	1.05–1.16	2.00	0.93	1.53–2.47	**0.00**
Relationship at home and with friends	Q7_WB	1–3	1.23	0.54	1.14–1.33	1.67	0.82	1.25–2.08	**0.01**
	Q8_WB	1–3	1.09	0.31	1.03–1.14	1.33	0.49	1.09–1.58	**0.00**
	Q9_WB	1–3	1.27	0.57	1.17–1.37	1.80	0.86	1.36–2.24	**0.00**
Enjoyment of school	Q10_WB	1–3	1.22	0.53	1.12–1.31	1.50	0.76	1.10–1.90	0.07
	Q11_WB	1–3	1.31	0.59	1.20–1.41	2.27	0.46	2.04–2.50	**0.00**
	Q12_WB	1–3	1.23	0.52	1.13–1.32	1.50	0.76	1.10–1.90	0.10
**Noise**									
Traffic disturbance	Q1_N	0–3	1.87	0.87	0.62–1.01	2.00	1.00	0.87–3.00	0.79
Siren disturbance	Q2_N	0–3	1.86	0.84	0.86–1.25	2.22	0.67	1.79–2.66	0.19
Disturbance from internal noises	Q3_N	0–3	1.79	0.83	0.93–1.31	2.09	0.54	1.77–2.41	0.18
Rain disturbance	Q4_N	0–3	1.74	0.75	0.92–1.29	1.56	0.73	1.08–2.03	0.47
Intensity and disturbance during silent task	Q5_N	1–3	1.38	0.63	1.27–1.49	1.73	0.88	1.29–2.18	0.11
	Q6_N	1–3	1.26	0.56	1.16–1.36	1.47	0.64	1.14–1.79	0.11
Intensity and disturbance during group activity	Q7_N	1–3	1.91	0.80	1.77–2.05	2.07	0.80	1.66–2.47	0.47
	Q8_N	1–3	1.72	0.79	1.58–1.86	1.93	0.70	1.58–2.29	0.24
Quality of voice	Q9_N	1–3	1.27	0.59	1.16–1.37	1.53	0.74	1.16–1.91	0.07
	Q10_N	1–3	1.50	0.65	1.39–1.62	2.20	0.86	1.76–2.64	**0.00**

*Mean (*M*), standard deviation (SD), and the 95% confidence interval (95% CI) values are provided and the *p*-values of significance for the differences between the two happiness conditions, according to the MWU test. Any statistically significant differences, with a *p*-value < 0.05, are reported in bold.*

**TABLE 8:** Subjective responses on the perceived well-being and noise disturbance of students in bad acoustics only, with the sample being grouped per happiness (i.e., happy and unhappy).

**Topic**	**ID**	**Scale**	**Happy subjects**			**Unhappy subjects**			**MWU *p*-value**
			**(*N* = 172)**			**(*N* = 15)**			
			***M***	**SD**	**95% CI**	***M***	**SD**	**95% CI**	
**Well-being**									
Self-esteem	Q1_WB	1–3	1.22	0.53	1.16–1.91	1.53	0.74	1.16–1.91	**0.03**
	Q2_WB	1–3	1.50	0.73	1.81–2.59	2.20	0.77	1.81–2.59	**0.00**
	Q3_WB	1–3	1.19	0.48	1.24–2.19	1.71	0.91	1.24–2.19	**0.01**
Emotional health	Q4_WB	1–3	1.33	0.59	1.21–1.86	1.53	0.64	1.21–1.86	0.12
	Q5_WB	1–3	1.28	0.57	1.29–2.18	1.73	0.88	1.29–2.18	**0.02**
	Q6_WB	1–3	1.15	0.41	1.82–2.71	2.27	0.88	1.82–2.71	**0.00**
Relationship at home and with friends	Q7_WB	1–3	1.24	0.57	1.25–2.22	1.73	0.96	1.25–2.22	**0.01**
	Q8_WB	1–3	1.12	0.39	1.18–2.02	1.60	0.83	1.18–2.02	**0.00**
	Q9_WB	1–3	1.24	0.47	1.53–2.47	2.00	0.93	1.53–2.47	**0.00**
Enjoyment of school	Q10_WB	1–3	1.35	0.70	1.55–2.59	2.07	1.00	1.55–2.59	**0.00**
	Q11_WB	1–3	1.30	0.54	2.21–2.73	2.47	0.52	2.21–2.73	**0.00**
	Q12_WB	1–3	1.33	0.61	2.20–3.00	2.62	0.77	2.20–3.00	**0.00**
**Noise**									
Traffic disturbance	Q1_N	0–3	1.94	0.83	0.00–0.44	1.00	0.00	/	0.05
Siren disturbance	Q2_N	0–3	1.90	0.77	0.00–0.94	1.50	1.00	0.52–2.48	0.28
Disturbance from internal noises	Q3_N	0–3	1.74	0.76	0.46–1.54	1.88	0.64	1.43–2.32	0.53
Rain disturbance	Q4_N	0–3	1.84	0.91	1.11–2.17	1.77	0.93	1.27–2.27	0.81
Intensity and disturbance during silent task	Q5_N	1–3	1.49	0.72	1.49–2.38	1.93	0.88	1.49–2.38	**0.04**
	Q6_N	1–3	1.50	0.76	1.33–2.14	1.73	0.80	1.33–2.14	0.19
Intensity and disturbance during group activity	Q7_N	1–3	2.12	0.79	2.14–2.66	2.40	0.51	2.14–2.66	0.22
	Q8_N	1–3	1.94	0.83	1.86–2.67	2.27	0.80	1.86–2.67	0.14
Quality of voice	Q9_N	1–3	1.23	0.53	1.14–1.79	1.47	0.64	1.14–1.79	0.05
	Q10_N	1–3	1.52	0.73	1.28–2.15	1.71	0.83	1.28–2.15	0.34

*Mean (*M*), standard deviation (SD), and the 95% confidence interval (95% CI) values are provided and the *p*-values of significance for the differences between the two happiness conditions, according to the MWU test. Any statistically significant differences, with a *p*-value < 0.05, are reported in bold.*

• Correlation matrix of the perceived well-being and noise disturbance scores of happy children with the classes and schools characteristics (Table 9);

**TABLE 9:** Correlation matrix of the perceived well-being and noise disturbance scores of the happy children with the class and the school characteristics (with MT being mother tongue, DV being district real estate value, TV being traffic volume, and AT being acoustic treatment) and the reverberation time and the background noise level in the empty classrooms, i.e., T20_e and L_N_sil_, respectively.

**ID**	**Question or parameter**	**Class and school characteristics**			
		**MT**	**DV**	**TV**	**AT**
MT	Mother tongue percentage	1			
DV	District real estate value		1		
TV	Traffic volume	−0.54	−0.22	1	
AT	Acoustic treatment		0.75	−0.27	1
T20_e (s)	Reverberation time in empty classroom	−0.31	−0.44	0.56	−0.63
L_N_sil_ (dBA)	Noise level in silence	−0.36		0.55	−0.35
Q1_WB	Proudness				
Q2_WB	Serenity			0.21	
Q3_WB	Fun				
Q4_WB	Holding many positive things about himself/herself				
Q5_WB	Gladness	−0.18			
Q6_WB	Cheerfulness				
Q7_WB	Relaxing at home		−0.18		
Q8_WB	Enjoyment with friends				
Q9_WB	Getting help from friends		−0.21		
Q10_WB	Pleasure of being at school				
Q11_WB	Safety at school				
Q12_WB	Fitting in at school				
Q1_N	Traffic disturbance			0.28	
Q2_N	Siren disturbance			0.22	
Q3_N	Disturbance from internal noises			0.21	
Q4_N	Rain disturbance				
Q5_N	Intensity during silent task	−0.20			
Q6_N	Disturbance during silent task	−0.19		0.16	
Q7_N	Intensity during group activity				
Q8_N	Disturbance during group activity				
Q9_N	Quality of the teacher’ voice				
Q10_N	Quality of the classmates’ voice				

*Spearman correlation coefficients are given for significant relationships with a *p*-value < 0.01.*

• Correlation matrix of the acoustic parameters (Table 10);

**TABLE 10:** Correlation matrix of the acoustic parameters measured in the classrooms.

	**Acoustic parameters**									
	**T20_e (s)**	**T20 (s)**	**L_N_sil_ (dBA)**	**L_N_gr_ (dBA)**	**L_S_REF_ (dBA)**	**mL_S_ (dBA/dd)**	**C50_ctr (dB)**	**C50_M (dB)**	**U50_ctr (dB)**	**U50_M (dB)**
T20_e (s)	1									
T20 (s)	0.86	1								
L_N_sil_ (dBA)	0.27		1							
L_N_gr_ (dBA)	0.64	0.48	0.49	1						
L_S___REF_ (dBA)	0.73	0.59	0.35	0.40	1					
mL_S_ (dBA/dd)	0.57	0.51			0.36	1				
C50_ctr (dB)	−0.84	−0.90		−0.36	−0.55	−0.46	1			
C50_M (dB)	−0.88	−0.96		−0.56	−0.48	−0.48	0.91	1		
U50_ctr (dB)	−0.71	−0.87	−0.36	−0.46	−0.65	−0.30	0.90	0.82	1	
U50_M (dB)	−0.84	−0.90	−0.37	−0.67	−0.62	−0.46	0.80	0.93	0.89	1

*Spearman correlation coefficients are given for significant relationships with a *p*-value < 0.01.*

• Correlation matrix of the perceived well-being and noise disturbance scores of happy children with the acoustic parameters (Table 11).

**TABLE 11:** Correlation matrix of the perceived well-being and noise disturbance scores for the happy children with the acoustic parameters.

**ID**	**Question**	**Acoustic parameters**								
		**T20**	**L_N_sil_**	**L_N_gr_**	**L_S_REF_**	**mL_S_**	**C50_ctr**	**C50_M**	**U50_ctr**	**U50_M**
		**(s)**	**(dBA)**	**(dBA)**	**(dBA)**	**(dBA/dd)**	**(dB)**	**(dB)**	**(dB)**	**(dB)**
Q1_WB	Proudness			−0.18						
Q2_WB	Serenity									
Q3_WB	Fun								−0.16	
Q4_WB	Hold many positive things about himself/herself									
Q5_WB	Gladness					0.18				
Q6_WB	Cheerfulness									
Q7_WB	Relax at home									
Q8_WB	Enjoinment with friends									
Q9_WB	Get help from friends									
Q10_WB	Pleasure of being at school									
Q11_WB	Safety at school									
Q12_WB	Fit in at school									
Q1_N	Traffic disturbance	0.30		0.33						
Q2_N	Siren disturbance									
Q3_N	Disturbance from internal noises	0.26								−0.22
Q4_N	Rain disturbance									
Q5_N	Intensity during silent task					0.28				−0.17
Q6_N	Disturbance during silent task					0.22	−0.17	−0.17	−0.16	−0.18
Q7_N	Intensity during group activity	0.16			0.18		−0.17		−0.24	−0.17
Q8_N	Disturbance during group activity								−0.19	
Q9_N	Quality of the teacher’s voice									
Q10_N	Quality of the classmates’ voice									

*Spearman correlation coefficients are given for significant relationships with a *p*-value < 0.01.*

Standard deviations in Tables 4–7 are quite high considering the 1–3 points scale of the adopted questionnaires. However, they are proportionally comparable to those reported in literature for children aged from 6–7 to 10–11 years in Dockrell and Shield (2004), and in the range 9–13 years in Brännström et al. (2017), which adopted 1–5 points scales.

As far as the correlation analysis is concerned, some of the correlation coefficients shown in Tables 10, 11 are low in absolute value, leading anyway to weak but statistically significant relationships according to the size of the samples.

### Influence of Bad Classroom Acoustics on Well-Being and Noise Disturbance for Happy and Unhappy Children

Table 4 shows higher mean values, which corresponds to worse conditions, in BA compared to GA, with significant differences according to the MWU Test, for the answers to the question Q12_WB related to the enjoyment of school, and particularly to the fitting in at school. The same occurs for the answers to the questions Q7_N, Q8_N, and Q9_N, which relate to noise disturbance during silence tasks, and to intensity and disturbance during group activity, respectively. The results highlight as noise in classrooms with higher reverberation, either from outdoor or indoor, can be perceived as more disturbing being more amplified by reverberation.

The same results that concern noise disturbance have also been found for happy students, as shown in Table 5, while Table 6 shows as for unhappy students the main problem in BA only concern serenity (Q2_WB), pleasances with himself or herself (Q5_WB) and fitting in at school (Q12_WB).

Tables 7, 8 show the comparison between happy and unhappy students’ subjective responses on the perceived well-being and noise disturbance, in good and bad classroom acoustics, respectively. Seven out of twelve well-being items have been judged significantly worse by unhappy students in GA, compared to eleven out of twelve in BA, thus suggesting as bad classroom acoustics determines less well-being that GA.

### Influence of Classes and School Characteristics on Well-Being and Noise Disturbance

Table 9 shows the most significant correlations between the perceived well-being and noise disturbance answers related to the happy children and the classes and schools characteristics. To this analysis’ aim, correlations were run between individual subjective responses (i.e., on perceived well-being and noise disturbance) and overall classes and schools characteristics, as specific objective data did not differ from pupil to pupil. Only Spearman correlation coefficients with *p*-values lower than 0.01 are shown. All the relationships are coherent among them, but the most interesting ones deserve some comments that are reported below:

• The percentage of Italian MT children is negative related to the traffic volume (TV), to the reverberation time in the empty classrooms, T20_e, and to the background noise level with the children in silence, L_N_sil_, thus showing as schools with a higher percentage of MT children have better acoustics. This is also confirmed by the lower perception of intensity and disturbance of noise in quiet (Q5_N and Q6_N, respectively), when the MT are higher. Moreover, MT is negatively related to the feeling of being pleased with themselves (Q5_WB), thus suggesting as a higher MT is related to higher well-being.
• The district real estate value (DV) is negatively related to the TV and to the acoustic parameters T20_e and L_N_sil_, and it is also positively related to the presence of AT. These results prove as richer districts allows for schools with better acoustics. DV is also negatively related to the goodness of the children’ s relationships at home and with friends (Q7_WB and Q9_WB), generally indicating that growing in poorer districts can bring to less well-being.
• High TV determines lower serenity and increases noise disturbance, as shown from the positive relationships of TV with the feeling of being serene (Q2_WB) and with the perception of disturbance of noise from outdoor and indoor (Q1_N, Q2_N, Q3_N, and Q6_N).
• Acoustic treatments is negatively related to the reverberation time in the empty classrooms, T20_e, and to the background noise level with the children in silence, L_N_sil_, as expected.

### Relationships Between Acoustic Parameters and Subjective Outcomes

#### Correlations Between Acoustic Parameters

Table 10 shows the correlations between the acoustic parameters measured in the classrooms. Only Spearman correlation coefficients with *p*-value less than 0.01 are shown. Very high relationships are shown between the acoustic quantities, as expected. Particularly most of the quantities are very well related to the reverberation time T20, both empty and occupied.

The parameters L_N_sil_, L_N_gr_, L_S___REF_, and mL_S_ are positive related to the reverberation time, that is when T20 is higher they are higher, as expected, and negatively related to C50 and U50. A very tight connection is shown between central and mean values of the quantities C50 and U50, thus bringing to the practical conclusion that only one measurement in the center of the room can well describe the behavior of the whole classroom in terms of speech intelligibility, as already shown by Puglisi et al. (2017). U50_ctr is also well related to C50_ctr, which suggests the use of only one quantity instead of two to represent speech intelligibility.

#### Correlations Between Acoustic Parameters and Well-Being and Noise Disturbance Scores

In order to choose among the most important objective quantities to be measured in a classroom it is useful to look at the results in Table 11, which shows the correlation matrix of the perceived well-being and noise disturbance scores for the happy children with the acoustic parameters. Most of the results are coherent and meaningful and can be useful to understand more about the relationships between environmental factors and children perception and behavior. The following interesting outcomes can be drawn:

• Generally, a positive relationship between disturbance from noise coming from outside the classroom, and in particular from traffic (Q1_N) and from adjacent rooms and corridor (Q3_N), and the noise level measured when the children were in silence, L_N___Sil_, has been found, well underling as poor sound insulation is related to higher sound levels in classrooms and consistent higher noise disturbance.
• The higher is the slope of the speech level along the central line of the classroom, mL_S_, or the lower is the slope when taken in absolute value, the higher is the perceived noise intensity and noise disturbance when the children are doing a task in silence (Q5_N and Q6_N, respectively). This is explainable considering that with higher values of speech slope the room is more reverberant and the speech level at the back of the classroom less clear, as well the noise is higher compared to less reverberant rooms.
• The higher the values of speech Clarity, C50, and Useful-to-detrimental-energy ratio, U50, both at the central position and average across the classroom, the lower are the noise intensity and disturbance scores Q3_N, Q5_N, Q6_N, Q7_N, and Q8_N. This means that good speech intelligibility corresponds to low noise disturbance both in the case of working in silence and in the case of group activity.
• As far as well-being is concerned, it is shown that the feeling of having fun is well related to higher values of speech intelligibility, in terms of U50_ctr, and the feeling of being happy of herself or himself is well related to lower values of mL_S_, i.e., in rooms with higher slope of L_S_, which means with less reverberation, children are happier with themselves.
• A particular remark is related to the parameter L_N_gr_, which is unexpected negative related to the well-being aspect “proudness of myself.” It means that the higher the level of noise during the group activity the higher is the proudness of the children. This result can be explained from the psychological point of view, as children were very happy while doing a lot of noise and they could be proud of having done it.

**FIGURE 2:**
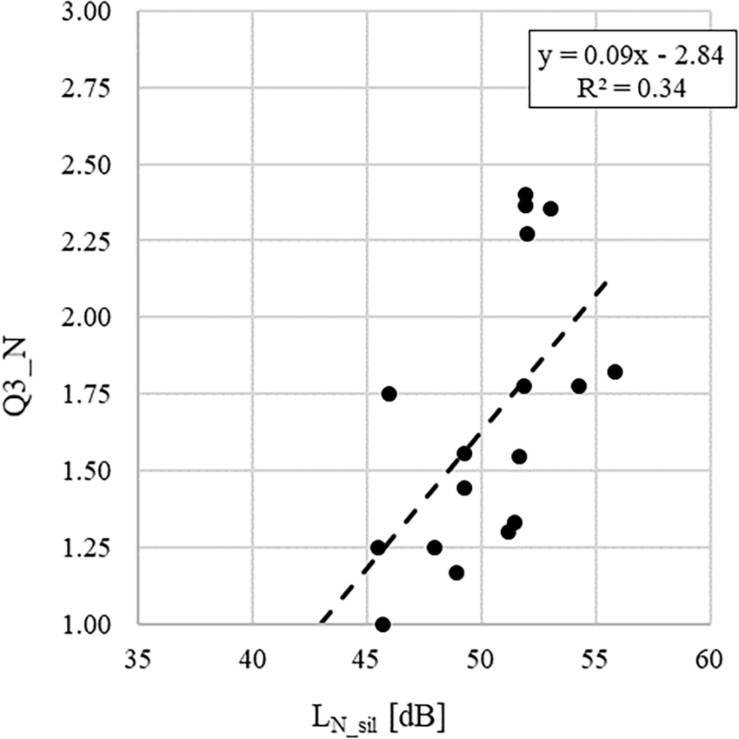
Linear regression graph showing the dependency of the average values across classes of the answers to the question Q3_N (i.e., “How much sounds of radios or recorders coming from other classrooms or from the corridor disturb you?”) on the noise level measured with children in silence, L_N_sil_.

**FIGURE 3:**
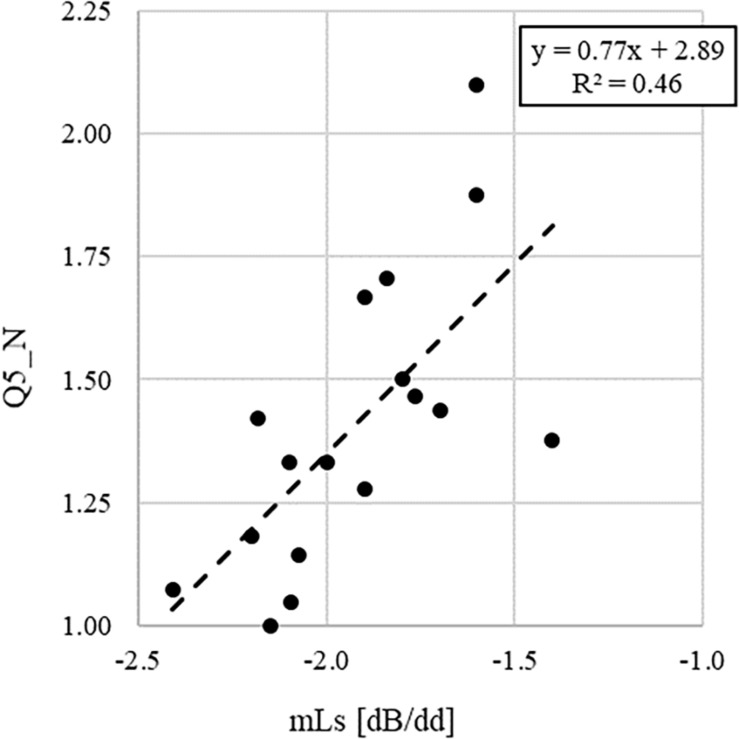
Linear regression graph showing the dependency of the average values across classes of the answers to the question Q5_N (i.e., “The noise present when you perform a task in silence seems you.”) on the slope of the voice signal, mL_S_.

With the aim to further clarify the relationships between subjective and objective parameters, Figures 2, 3 show, as example, the regression lines between the average values across classes of the answers to the questions Q3_N and Q5_N and the acoustic parameters L_N_sil_ and mL_S_, respectively. These relationships have been chosen as they show two of the highest correlation coefficients in Table 11.

## Discussion

In-field studies on the effects of classroom acoustics on the perception of noise disturbance are only a few in the available literature. Furthermore, no study has investigated the perception of well-being at school and its relationships with acoustics. The present study has aimed at investigating three main aspects that are lacking in the available literature so far: (i) the influence of bad classroom acoustics on perceived well-being and noise disturbance for happy and unhappy children; (ii) the influence of class and school characteristics on perceived well-being and noise disturbance; (iii) the relationships between classroom acoustic parameters and perceived well-being and noise disturbance scores.

### Role of Bad Classroom Acoustics on Well-Being and Noise Disturbance for Happy and Unhappy Children

The classrooms involved in the study are representative of the typical acoustic quality available in the majority of Italian schools, with reverberation times under occupied conditions ranging from 0.5 to 1.4 s. Therefore, they could be grouped under GA and BA labels depending on their performance in agreement with recent studies, which suggest an optimal reverberation time range between 0.5 and 0.8 s to account for both speaking and listening premises at the same time. Based on the GA and BA clustering of classrooms, the data from subjective questionnaires administrated to the pupils were analyzed to understand the extent to which GA and BA have an influence on the perception of well-being and noise disturbance at school. The presented results highlight that outdoor and indoor noise in BA is perceived as more disturbing than in GA, being significantly amplified by the higher reverberation. These outcomes corroborate previous studies that proved outdoor and indoor noise sources to be the most annoying for children of grades 2 and 6 (Dockrell and Shield, 2004) and high school students (Astolfi and Pellerey, 2008), respectively.

This study has shown that the 90.8% of the involved pupils reported to be happy. The subgroup of 9.2% of unhappy pupils has shown that the significant detrimental effect of BA was on the perception of serenity, self-pleasances, and fitting in at school, and not on increased noise disturbance. Overall, unhappy pupils have judged worse a higher number of well-being items in BA compared to GA, confirming the need of GA quality in classrooms to enhance well-being at school. This is consistent with a previous study by Klatte and Hellbrück (2010), who found that children attending classes under BA conditions judged their relationships to their peers and teachers less positively than did children from classrooms with GA.

### Influence of Classes and School Characteristics on Well-Being and Noise Disturbance Perception

School and classroom characteristics need to be accounted for in a study that investigates the subjective perception as it may be influenced by the subjects’ background, origins, and features. To the aim of the present work, schools and classrooms had to present different characteristics in order to provide objective outcomes that were not affected by formal bias. Therefore, objective information was collected that consisted in student and building features, the presence of AT, the year of construction, and the TV.

In classrooms with better acoustics, a higher percentage of mother-tongue-speaking children was present. Such a result was corroborated by the lower perception of noise intensity and disturbance in quiet. Moreover, the students’ feature of being speakers of the MT resulted in a higher feeling of being pleased with themselves. These outcomes suggest that being speakers of the MT is a child characteristic that brings more chances of attending classes in GA, being less disturbed, and having more well-being.

As far as school features are concerned, richer districts presented better acoustic conditions, resulting in turn in the fact that richer districts also revealed a greater perception of well-being.

Higher TVs were found to determine lower serenity and increase in noise disturbance. This outcome agrees with many studies, among which Klatte and Hellbrück (2010), Crombie et al. (2011), and Lim et al. (2018), who proved a significant association between noise exposure to noise and high levels of noise annoyance, as well as decreased well-being, but all the studies took into account children older than 6–7 years old and were mostly based on questionnaires that were not filled in directly by children at school.

### Relationships Between Classroom Acoustic Parameters and Well-Being and Noise Disturbance Scores

Overall, very high relationships were found between the acoustic quantities, corroborating past results (Bradley, 1986; Sato and Bradley, 2008; Yang and Bradley, 2009). The perceived noise intensity and disturbance were found to be strongly positively related to the slope of speech level along a classroom’s main axis (mL_S_) and strongly negatively related to speech clarity (C50) and the ratio of useful to detrimental energy (U50), both at the central position and on average across the classroom. These coherent outcomes are dependent on the reverberation time, which directly affects the uniformity of the sound energy in the room and increases the noise level: the higher is the reverberation time in the room, the higher is the speech slope along the main axis (or lower when it is considered in absolute values) and the lower are the values of speech clarity and the ratio of useful to detrimental energy. Much should then be done in the acoustic design of classrooms to guarantee optimal distribution of parameters across all the listening positions. Promising results are achieved by combining the use of absorptive and diffusive surfaces (Choi, 2014), even though the perceptive effect of diffusive linings is still under research (Shtrepi et al., 2015, 2016, 2017).

The very high correlation between speech clarity and the ratio of useful to detrimental energy and the tight connection between central and mean values suggest the adoption of only one of the two indexes measured in the central position of the room to evaluate the classroom speech intelligibility. This outcome is in agreement with the results found by Puglisi et al. (2017), who suggested to measure clarity in the middle of the classroom to characterize its average behavior in terms of speech intelligibility.

U50 in the central position of the classroom was also strongly related to the feeling of having fun. A strong positive relationship was found for mL_S_ and the feeling of children of being happy with themselves; that is, the higher the slope (or the lower in absolute value), the higher is the feeling of being unpleased with oneself. To the authors’ knowledge, no studies are available in literature that considered the influence of speech intelligibility parameters and the effect of reverberation on the perceived well-being of young children. Anyway, the obtained results on the influence of acoustics on well-being at school are in line with those from Wålinder et al. (2007), who found that noise can be considered as a risk factor for the children’s well-being in the school environment because it has primary consequences on the increased prevalence of symptoms of fatigue and headache.

Furthermore, the noise levels measured inside the classrooms were found to be significantly correlated with the perception of outdoor noise sources, in particular from traffic and from adjacent rooms and corridor. This relationship supports the need of an accurate acoustic design of classrooms with a focus on the enhancement of sound insulation as suggested in Secchi et al. (2017), as it is related to higher sound levels in classrooms and consistent with higher noise disturbance.

### Strengths and Limitations of the Study

The present study confirms the association between bad classroom acoustics and noise disturbance for children at school. The main strength of the study is that the acoustic parameters needed to discriminate BA and GA were measured accurately, on the basis of referred protocols, and considers all the classroom acoustic aspects. In addition, this is the first study that examines the combined effect of noise disturbance and well-being perception of first graders at school. On the one side, the wide range of classroom acoustic conditions chosen for the study, ranging from 0.5 to 1.4 s of reverberation time, has ensured the covering of most of the current classrooms’ features. On the other side, the school characteristics sufficiently covered most of the school buildings of a typical town.

There are also several limitations in the present study. The selection of the schools depended on their willingness to participate in the research, which was part of a wider study on the assessment and strengthening of the reading abilities of first graders (Astolfi et al., 2018b). Furthermore, the teachers of first graders volunteered to participate in the study, probably due to an already existing interest in the strengthening of the reading abilities of the pupils. The influence of the acoustic environment on the children’s abilities was only proposed as a secondary outcome to the teachers. All the invited children of the classrooms (with the consent of their legal guardians) agreed to participate in the study.

Well-being was measured using an Italian translation of the questionnaire developed by Sabri et al. (2015), suitable for young people with SENs. It was selected as it was validated for children aged 6–7 years old and because it was especially designed for well-being investigations at school. The questionnaire included many items which may have affected their completion rate, especially for first graders. The items were *a priori* explained to the children by an experimenter, who was always the same, but this could have biased the answers, involuntarily.

In addition, the questionnaires filled in by children with hearing impairment (HI) or with SENs identified by the teachers were excluded, and these data have not been used for further analyses. It is well known that children with HI or SENs are more susceptible to the adverse effects of BA, and future studies need to survey this fragile group of pupils. Moreover, because this is a cross-sectional study, the causality of the association cannot be univocally determined. To investigate temporal changes in noise disturbance and well-being also for particular groups of children, as well as to infer causality, longitudinal studies are needed based on a larger population. Additional information on parent’s and family conditions, both related to economic and educational issues and on well-being and noise disturbance perceived at home, should have been acquired.

An unexpected result consisted in the strong relationship that was found between L_N_gr_ and the well-being aspect “proudness of myself,” as it was found that pupils were prouder of themselves as the level due to activity noise was higher in the classroom. This outcome should be deepened from a psychological point of view rather than an acoustic point of view, as it may be that due to the excitement of children when they were asked to be noisy, the measurement condition was biased. Thus, a proposal for the future is to measure other noise levels in the classrooms, related to real noise situations during lessons; however, it is needed to adopt an effective measurement procedure that avoids external influences on the behavior of pupils.

## Conclusion

In the present study, subjective outcomes on the assessment of perceived well-being and noise disturbance at school for first graders were related to classroom acoustics characteristics and to classes and schools features.

The findings of this pilot study, which involved 326 first graders in their own classrooms, suggest that long reverberation times, which are associated with poor classroom acoustics as they generate higher noise levels and degrade speech intelligibility, bring pupils to a reduced perception of having fun and being happy with themselves. Furthermore, bad classroom acoustics is also related to an increased perception of noise intensity and disturbance, particularly in the case of traffic noise and noise from adjacent school environments.

Finally, an analysis of the perception of well-being and noise disturbance depending on the self-judgment of pupils on being happy or unhappy was performed. Happy pupils reported a higher perception of noise disturbance under bad classroom acoustic conditions, whereas unhappy pupils reported only complaints in bad classroom acoustics with respect to the perception of pleasances with himself or herself and of fitting in at school.

## Data Availability Statement

The datasets generated for this study are available on request to the corresponding author.

## Ethics Statement

The studies involving human participants were reviewed and approved by the Ethics Committee of Politecnico di Torino. Written informed consent to participate in this study was provided by the participants’ legal guardian/next of kin.

## Author Contributions

AA and GP contributed in the conception and design of the study. AA, GP, AP, SM, and GM participated in the in-field measurements and surveys. SM and GM organized the database. GP and AP drafted the “Introduction” section. GM and GP drafted the “Materials and Methods” section. AA, FP, and SM performed the statistical analysis. AA, GP, GM, and AP wrote the first draft of the manuscript. AA directed the research, wrote the “Results,” “Discussion,” and “Conclusion” sections, and drafted the final version of the manuscript. TS provided research assistance and commented on earlier versions of the manuscript. All authors contributed to manuscript revision and read and approved the submitted version.

## Conflict of Interest

The authors declare that the research was conducted in the absence of any commercial or financial relationships that could be construed as a potential conflict of interest.

## References

[B1] ANSI S3.5, (1997). *Methods for Calculation of the Speech Intelligibility Index.* New York, NY: Acoustical Society of America.

[B2] Astolfi A. Pellerey F. (2008). Subjective and objective assessment of acoustical and overall environmental quality in secondary school classrooms. *J. Acoust. Soc. Am.* 123 163–173. 10.1121/1.2816563 18177148

[B3] Astolfi A. Bottalico P. Accornero A. Garzaro M. Nadalin J. Giordano C. (2012a). “Relationship between vocal doses and voice disorders on primary school teachers,” in *Proceedings of the Conference on Noise Control – EuroNoise2012*, Prague.

[B4] Astolfi A. Bottalico P. Barbato G. (2012b). Subjective and objective speech intelligibility investigations in primary school classrooms. *J. Acoust. Soc. Am.* 131 247–257. 10.1121/1.3662060 22280588

[B5] Astolfi A. Carullo A. Pavese L. Puglisi G. E. (2015). Duration of voicing and silence periods of continuous speech in different acoustic environments. *J. Acoust. Soc. Am.* 137 565–579. 10.1121/1.4906259 25697991

[B6] Astolfi A. Castellana A. Carullo A. Puglisi G. E. (2018a). Uncertainty of speech level parameters measured with a contact-sensor-based device and a headworn microphone. *J. Acoust. Soc. Am.* 143 EL496–EL502. 10.1121/1.5042761 29960427

[B7] Astolfi A. Castellana A. Puglisi G. E. Fugiglando U. Carullo A. (2019). Speech level parameters in very low and excessive reverberation measured with a contact-sensor-based device. *J. Acoust Soc. Am.* 145 2540–2551. 10.1121/1.5098942 31046351

[B8] Astolfi A. Corrado V. Griginis A. (2008). Comparison between measured and calculated parameters for the acoustical characterization of small classrooms. *Appl. Acoust.* 69 966–976. 10.1016/j.apacoust.2007.08.001

[B9] Astolfi A. Puglisi G. E. Minelli G. Shtrepi L. Prato A. Sacco T. (2018b). “Effects of classroom acoustics on potential struggling first-grade readers,” in *Proceedings of the Conference on Noise Control – EuroNoise2018*, Crete.

[B10] Bottalico P. Astolfi A. (2012). Investigations into vocal doses and parameters pertaining to primary school teachers in classrooms. *J. Acoust. Soc. Am.* 131 2817–2827. 10.1121/1.3689549 22501060

[B11] Bottalico P. Astolfi A. Hunter E. J. (2017a). Teachers’ voicing and silence periods during continuous speech in classrooms with different reverberation times. *J. Acoust. Soc. Am.* 141 El26–El31. 10.1016/S0892-1997(03)00033-X 28147593 PMC5392096

[B12] Bottalico P. Graetzer S. Astolfi A. Hunter E. J. (2017b). Silence and voicing accumulations in Italian primary school teachers with and without voice disorders. *J. Voice* 31 260.e11–260.e20. 10.1016/j.jvoice.2016.05.009 27316793 PMC5156594

[B13] Bottalico P. Ipsaro Passione I. Astolfi A. Carullo A. Hunter E. J. (2018). Accuracy of the quantities measured by four vocal dosimeters and its uncertainty. *J. Acoust. Soc. Am.* 143 1591–1602. 10.1121/1.5027816 29604673 PMC5864503

[B14] Bradley J. S. (1986). Speech intelligibility studies in classrooms. *J. Acoust. Soc. Am.* 80 846–854. 10.1121/1.393908 3760338

[B15] Bradley J. S. Reich R. D. Norcross S. G. (1999). On the combined effects of signal-to-noise ratio and room acoustics on speech intelligibility. *J. Acoust. Soc. Am.* 106 1820–1828. 10.1121/1.427932 10530010

[B16] Brännström K. J. Johansson E. Vigertsson D. Morris D. J. Sahlén B. Lyberg-Åhlander V. (2017). How children perceive the acoustic environment of their school. *Noise Health* 19 84–94. 10.4103/nah.NAH-33-16 29192618 PMC5437757

[B17] Calosso G. Puglisi G. E. Astolfi A. Castellana A. Carullo A. Pellerey F. (2017). A one-school year longitudinal study of secondary school teachers’ voice parameters and the influence of classroom acoustics. *J. Acoust. Soc. Am.* 142 1055–1066. 10.1121/1.4998707 28863620

[B18] Cardon G. Campbell J. Sharma A. (2012). Plasticity in the developing auditory cortex: evidence from children with sensorineural hearing loss and auditory neuropathy spectrum disorder. *J. Am. Acad. Audiol.* 23 396–495. 10.3766/jaaa.23.6.3 22668761 PMC3733172

[B19] Carullo A. Vallan A. Astolfi A. (2013). “A low-cost platform for voice monitoring,” in *Proceedings of the International Instrumentation and Measurement Technology Conference (I2MTC)*, Minneapolis.

[B20] Castellana A. Carullo A. Astolfi A. Puglisi G. E. Fugiglando U. (2017). Intra-speaker and inter-speaker variability in speech sound pressure level across repeated readings. *J. Acoust. Soc. Am.* 141 2353–2363. 10.1121/1.4979115 28464626

[B21] Choi Y.-J. (2014). The application of diffusers for classroom acoustical design. *Noise Vib. Worldwide* 45 8–16. 10.1260/0957-4565.45.5.8

[B22] Crombie R. Clark C. Stansfeld S. A. (2011). Environmental noise exposure, early biological risk and mental health in nine to ten years old children: a cross-sectional field study. *Environ. Health* 10 1–8. 10.1186/1476-069X-10-39 21569605 PMC3117762

[B23] Croux C. Dehon C. (2010). Influence functions of the spearman and kendall correlation measures. *Statist. Methods Appl.* 19 497–515. 10.1007/s10260-010-0142-z

[B24] Di Blasio S. Shtrepi L. Puglisi G. E. Astolfi A. (2019). A cross-sectional survey on the impact of irrelevant speech noise on annoyance, mental health and well-being, performance and occupants’ behavior in shared and open-plan offices. *Int. J. Environ. Res. Public Health* 16:280. 10.3390/ijerph16020280 30669442 PMC6351961

[B25] Diener E. (2006). Guidelines for national indicators of subjective wellbeing and ill-being. *J. Happiness Stud.* 7 397–404. 10.1007/s10902-006-9000-y

[B26] Din 18041. (2016). *Hörsamkeit in Räumen–Anforderungen Empfehlungen und Hinweise Für Die Planung (Acoustic Quality in Rooms–Specifications and Instructions for the Room Acoustic Design).* Berlin: German Institute for Standardisation.

[B27] Dockrell J. E. Shield B. (2004). Children’s perceptions of their acoustic environment at school and at home. *J. Acoust. Soc. Am.* 115 2964–2973. 10.1121/1.1652610 15237821

[B28] Dreger S. Meyer N. Fromme H. Bolte G. Hendrowarsito L. Frieß-Hesse T. (2015). Environmental noise and incident mental health problems: a prospective cohort study among school children in Germany. *Environ. Res.* 143 49–54. 10.1016/j.envres.2015.08.003 26433757

[B29] Epstein J. L. McPartland J. M. (1976). The concept and measurement of the quality of school life. *Am. Educ. Res. J.* 13 15–30. 10.3102/00028312013001015

[B30] Evans G. W. Lercher P. Meis M. Ising H. Kofler W. W. (2001). Community noise exposure and stress in children. *J. Acoust. Soc. Am.* 109 1023–1027. 10.1121/1.1340642 11303916

[B31] Forns J. Dadvand P. Foraster M. Alvarez-Pedrerol M. Rivas I. López-Vicente M. (2016). Traffic-Related air pollution, noise at school, and behavioral problems in Barcelona schoolchildren: a cross-sectional study. *Environ. Health Perspect.* 124 529–535. 10.1289/ehp.1409449 26241036 PMC4829987

[B32] Goodman R. (2001). Psychometric properties of the strengths and difficulties questionnaire (SDQ). *J. Am. Acad. Child Adolesc. Psychiatry* 40 1337–1345. 10.1097/00004583-200111000-00015 11699809

[B33] Guski R. Felscher-Suhr U. Schuemer R. (1999). The concept of noise annoyance: how international experts see it. *J. Sound Vib.* 223 513–527. 10.1006/jsvi.1998.2173

[B34] Haines M. M. Stansfeld S. A. Job R. F. Berglund B. Head J. (2001). Chronic aircraft noise exposure, stress responses, mental health and cognitive performance in school children. *Psychol Med.* 31 265–277. 10.1017/S0033291701003282 11232914

[B35] Hascher T. (2008). Quantitative and qualitative research approaches to assess student well-being. *Int. J. Educ. Res.* 47 84–96. 10.1016/j.ijer.2007.11.016

[B36] Hjortebjerg D. Andersen A. M. N. Christensen J. S. Ketzel M. Raaschou-Nielsen O. Sunyer J. (2016). Exposure to road traffic noise and behavioural problems in 7-year-old children: a cohort study. *Environ. Health Perspect.* 124 228–234. 10.1289/ehp.1409430 26126294 PMC4749080

[B37] ISO 18233, (2006). *Acoustics: Application of New Measurement methods in Building and Room Acoustics.* Geneva: International Organization for Standardization.

[B38] ISO 3382-1, (2009). *Acoustics - Measurement of Room Acoustic Parameters – Part 1: Performance Spaces.* Genève: International Organization for Standardization.

[B39] ISO 3382-2, (2008). *Acoustics: Measurement of Room Acoustic Parameters–Reverberation Time in Ordinary Rooms.* Geneva: International Organization for Standardization.

[B40] ISO 9921, (2015). *Ergonomics: Assessment of Speech Communication.* Geneva: International Organization for Standardization.

[B41] Keyes C. L. (ed.) (2013). *Mental Wellbeing. International Contributions to the Study of Positive Mental Health.* Georgia: Springer.

[B42] Klatte M. Hellbrück J. (2010). Effects of classroom acoustics on performance and well-being in elementary school children: a field study. *Environ. Behav.* 42 659–692. 10.1177/0013916509336813

[B43] Klatte M. Bergström K. Lachmann T. (2013). Does noise affect learning? A short review on noise effects on cognitive performance in children. *Front. Psychol.* 4:578. 10.3389/fpsyg.2013.00578 24009598 PMC3757288

[B44] Konu A. Alanen E. Lintonen T. Rimpelä M. (2002). Factor structure of the school well-being model. *Health Educ. Res.* 17 732–742. 10.1093/her/17.6.732 12507348

[B45] Lim J. Kweon K. Kim H. W. Cho S. W. Park J. Sim C. S. (2018). Negative impact of noise and noise sensitivity on mental health in childhood. *Noise Health* 20 199–211. 10.4103/nah.NAH-9-18 30516173 PMC6301087

[B46] Minelli G. Astolfi A. Puglisi G. E. Murgia S. Shtrepi L. Prato A. (2019). “Measuring classroom acoustics with a systematic approach,” in *Proceedings of Internoise 2019*, Madrid.

[B47] OICT, (2019). *Turin Real Estate Market Observatory.* Available at: http://www.oict.polito.it/en/ (accessed May, 2019).

[B48] Pelegrín-García D. Brunskog J. Rasmussen B. (2014). Speaker-oriented classroom acoustics design guidelines in the context of current regulations in European countries. *Acta Acust. Acust.* 100 1073–1089. 10.3813/AAA.918787

[B49] Prodi N. Visentin C. Feletti A. (2013). On the perception of speech in primary school classrooms: Ranking of noise interference and of age influence. *J. Acoust. Soc. Am.* 133 255–268. 10.1121/1.4770259 23297900

[B50] Puglisi G. E. Astolfi A. Cantor Cutiva L. C. Carullo A. (2015a). “Assessment of indoor ambient noise level in school classrooms,” in *Proceedings of the Conference on Noise Control – EuroNoise2015*, Maastricht.

[B51] Puglisi G. E. Cantor Cutiva L. C. Astolfi A. Carullo A. (2017). Four-day-follow-up study on the voice monitoring of primary school teachers: relationships with conversational task and classroom acoustics. *J. Acoust. Soc. Am.* 141 441–452. 10.1121/1.4973805 28147558

[B52] Puglisi G. E. Prato A. Sacco T. Astolfi A. (2018). Influence of classroom acoustics on the reading speed: a case study on Italian second-graders. *J. Acoust. Soc. Am.* 141 EL144–EL149. 10.1121/1.5051050 30180687

[B53] Puglisi G. E. Warzybok A. Hochmuth S. Visentin C. Astolfi A. Prodi N. (2015b). An Italian matrix sentence test for the evaluation of speech intelligibility in noise. *Int. J. Audiol.* 54 44–50. 10.3109/14992027.2015.1061709 26371592

[B54] Riley K. G. McGregor K. K. (2012). Noise hampers children’s expressive word learning. *Lang. Speech Hear. Serv. Sch.* 43 325–337. 10.1044/0161-1461 22411494 PMC3641792

[B55] Rudner M. Lyberg-Åhlander V. Brännström J. Nirme J. Pichora-Fuller M. K. Sahlén B. (2018). Listening comprehension and listening effort in the primary school classroom. *Front. Psychol.* 9:1193. 10.3389/fpsyg.2018.01193 30050489 PMC6052349

[B56] Ryff C. D. Singer B. (1998). The contours of positive human health. *Psychol. Inq.* 9 1–28. 10.1207/s15327965pli0901-1

[B57] Sabri F. Rotheroe A. Kazimirski A. (2015). *Measuring the Well-Being of Young People with Special Educational Needs.* London: New Philanthropy Capital.

[B58] Sato H. Bradley J. S. (2008). Evaluation of acoustical conditions for speech communication in working elementary school classrooms. *J. Acoust. Soc. Am.* 123 2064–2077. 10.1121/1.2839283 18397014

[B59] Secchi S. Astolfi A. Calosso G. Casini D. Cellai G. Scamoni F. (2017). Effect of outdoor noise and façade sound insulation on indoor acoustic environment of Italian schools. *Appl. Acoust.* 126 120–130. 10.1016/j.apacoust.2017.05.023

[B60] Shield B. M. Dockrell J. (2008). The effects of environmental and classroom noise on the academic attainments of primary school children. *J. Acoust. Soc. Am.* 123 133–144. 10.1121/1.2812596 18177145

[B61] Shtrepi L. Astolfi A. D’antonio G. Guski M. (2016). Objective and perceptual evaluation of distance-dependent scattered sound effects in a small variable-acoustics hall. *J. Acoust. Soc. Am.* 140 3651–3662. 10.1121/1.4966267 27908082

[B62] Shtrepi L. Astolfi A. Pelzer S. Vitale R. Rychtáriková M. (2015). Objective and perceptual assessment of the scattered sound field in a simulated concert hall. *J. Acoust. Soc. Am.* 138 1485–1497. 10.1121/1.4929743 26428786

[B63] Shtrepi L. Astolfi A. Puglisi G. E. Masoero M. C. (2017). Effects of the distance from a diffusive surface on the objective and perceptual evaluation of the sound field in a small simulated variable-acoustics hall. *Appl. Sci.* 224 1–23. 10.3390/app7030224

[B64] Sigel S. Castellan N. J. (1988). *Non Parametric Statistics for the Behavioral Sciences*, 2nd Edn, New York, NY: McGraw-Hill.

[B65] Stansfeld S. A. Clark C. Cameron R. M. Alfred T. Head J. Haines M. M. (2009). Aircraft and road traffic noise exposure and children’s mental health. *J. Environ. Psychol.* 29 203–207. 10.1016/j.jenvp.2009.01.002

[B66] Stansfeld S. A. Haines M. M. Burr M. Berry B. Lercher P. (2000). A review of environmental noise and mental health. *Noise Health* 2 1–8. 12689457

[B67] Tobia V. Greco A. Steca P. Marzocchi G. M. (2019). Children’s wellbeing at school: a multi-dimensional and multi-informant approach. *J. Happiness Stud.* 20 841–861. 10.1007/s10902-018-9974-2

[B68] UK Department of Transport (2012). Available ast: https://www.gov.uk/government/publications/guidance-on-road-classification-and-the-primary-route-network (accessed October 11, 2019).

[B69] UNI 11367 (2010). “Acustica in edilizia - classificazione acustica delle unità immobiliari - procedura di valutazione e verifica in opera,” in *Building Acoustics - Acoustic Classification of Living Units - Evaluation and in Field Verification procedures.* Milan, Italy: Ente Italiano di Normazione.

[B70] Wålinder R. Gunnarsson K. Runeson R. Smedje G. (2007). Physiological and psychological stress reactions in relation to classroom noise. *Scand. J. Work Environ. Health* 33 260–266. 10.5271/sjweh.1141 17717617

[B71] Winblad U. Dudley E. (1997). *Primary School Physical Environment and Health: WHO Global School Health Initiative.* Geneva: World Health Organization.

[B72] World Health Organisation [WHO] (2014). Available at: https://www.who.int/features/factfiles/mental_health/en/ (accessed May, 2019).

[B73] WSP (2015). *Acoustic Design in Schools: Performance Standards Building Bulletin, BB93.* London: Educational Funding Agency.

[B74] Yang W. Bradley J. S. (2009). Effects of room acoustics on the intelligibility of speech in classrooms for young children. *J. Acoust. Soc. Am.* 125 922–933. 10.1121/1.3058900 19206869

